# Specialized abdominoscrotal hydrocele: Case report (CARE-compliant)

**DOI:** 10.1097/MD.0000000000042974

**Published:** 2025-06-20

**Authors:** Ti-Xue Wang, Ya-Long Ma, Nuan Han, Lin Feng, Chong-Fang Zhang

**Affiliations:** a Department of Pediatric Surgery, Affiliated Hospital of Jining Medical University, Jining, Shandong Province, China; b Department of Clinical Medicine, Jining Medical University, Jining, Shandong Province, China.

**Keywords:** abdominoscrotal hydrocele, ASH, diagnosis, pathogenesis, treatment

## Abstract

**Rationale:**

Abdominoscrotal hydrocele (ASH) is a rare condition characterized by fluid-filled masses in the inguinal scrotal and abdominal components, and the understanding of this condition, especially its pathogenesis, remains incompletely transparent.

**Patient concerns:**

The child, a 2-year-old boy, 4 days ago, no apparent cause of paroxysmal abdominal pain during the constipation symptoms, self-defecation after abdominal pain relief, occasional mild cough, no nausea and vomiting.

**Diagnoses:**

“Abdominal cavity occupation.”

**Interventions:**

Laparoscopic exploration, the most prominent position of the cyst was opened with tissue scissors to cut the cyst wall extensively, the intra-abdominal purse-string suture was closed to close the internal ring opening on the affected side and tied with a knot.

**Outcomes:**

The child had no postoperative discomfort and was discharged from the hospital with no abnormalities on regular follow-ups and had returned to normal.

**Lessons:**

We reported a more unusual case with specific clinical manifestations that helped us to understand abdominoscrotal hydrocele further, and we conducted a systematic review of the relevant literature to provide an overview of the clinical manifestations, pathogenesis, diagnosis and treatment.

## 1. Introduction

Abdominoscrotal hydrocele (ASH) is a rare clinical condition, occurring in 0.4% to 3.1% of all cases of syringomyelia. The pathogenesis of ASH is still not fully understood.^[1]^ ASH is characterized by a gourd-shaped syringomyelia that extends through the scrotum and into the abdominal cavity. Its clinical manifestations and physical examination are similar to those of conventional syringomyelia. Still, its physical examination reveals a sizeable tense syringomyelia and an abdominal mass in a crossover pattern, that is, the “bouncing ball sign,”^[2]^ and an inguinal scrotal ultrasound reveals a cystic scrotal mass that extends through the inguinal canal and into the abdominal cavity. ASH seldom subsides spontaneously, and surgical intervention is required. To prevent the recurrence of ASH, traditionally, the inguinal, scrotal or combined approach is often used to remove altogether the intra-abdominal portion, which is extensive and traumatic and may result in complications such as vascular injury, spermatic ureteral injury, and testicular atrophy. There are attempts to use laparoscopic treatment of ASH, including laparoscopic techniques to resect part of the cystic wall with simultaneous repair of the internal circumferential orifice and laparoscopic cyst opening combined with single-site sphincter high ligation for ASH.

## 2. Case information

**Figure 1.**
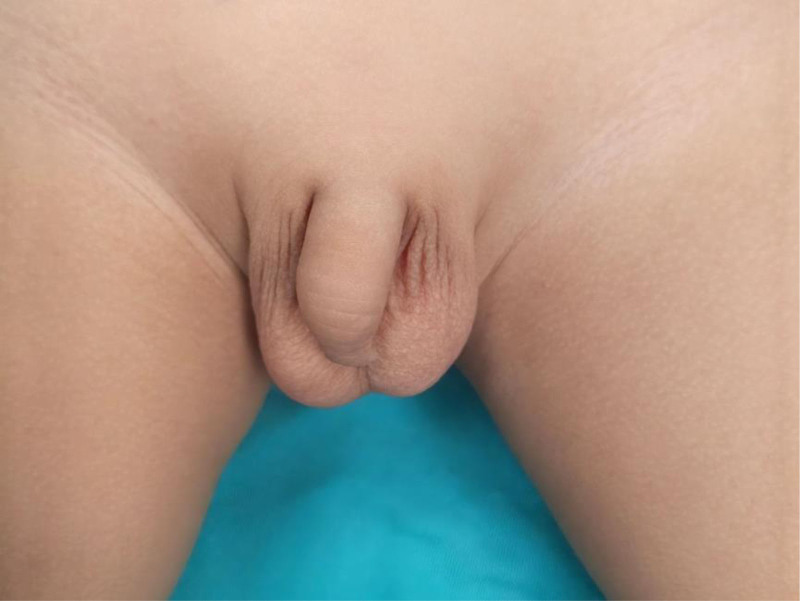
Bilateral inguinal scrotal area without abnormality.

The child, a 2-year-old boy, 4 days ago, no apparent cause of paroxysmal abdominal pain during the constipation symptoms, self-defecation after abdominal pain relief, occasional mild cough, no nausea and vomiting, the family did not pay attention, 3 days ago, paroxysmal severe abdominal pain, accompanied by fecal intractability, was consulted in the local hospital, color ultrasound examination suggests that: abdominal mass, given kestrel enemas, recommended that the child go to the higher level of hospitals, for further diagnosis and treatment, the family then came to our hospital outpatient clinic, in our hospital color ultrasound examination suggests that: right lower abdominal cystic mass; lymphatic origin mass? To seek further diagnosis and treatment, the family came to our outpatient clinic, where an ultrasound examination revealed a cystic mass in the right lower abdomen and a lymphatic mass of lymphatic origin. Further examination was recommended when necessary. The outpatient physician combined the child’s physical signs and auxiliary examination and admitted the patient to the hospital with “abdominal cavity occupation.” Since the onset of the disease, the child had a clear mind, typical spirit, regular diet, normal sleep, normal urination and defecation, no significant change in weight, and regular physical strength. Specialized examination: flat abdomen, no gastrointestinal type and peristaltic wave, soft abdomen, light pressure pain in the right lower abdomen, no rebound pain, no mobile turbidities, no apparent mass, bowel sound 2 times/min, no rash and bleeding in both lower limbs, and no abnormality in the bilateral inguinal scrotal area (Fig. 1).

Upon admission, a plain computed tomography (CT) scan and enhanced scan (dual-source CT) of the whole abdomen were performed, suggesting: 1. Irregular cystic low-density shadow in the right pelvis: a lymphatic cyst could be considered, and MR was recommended for further examination. 2. The right inguinal hernia was not excluded. To further clarify the nature of the mass, laparoscopic surgery was performed after obtaining the family’s consent.

## 3. Surgical procedures

**Figure 2.**
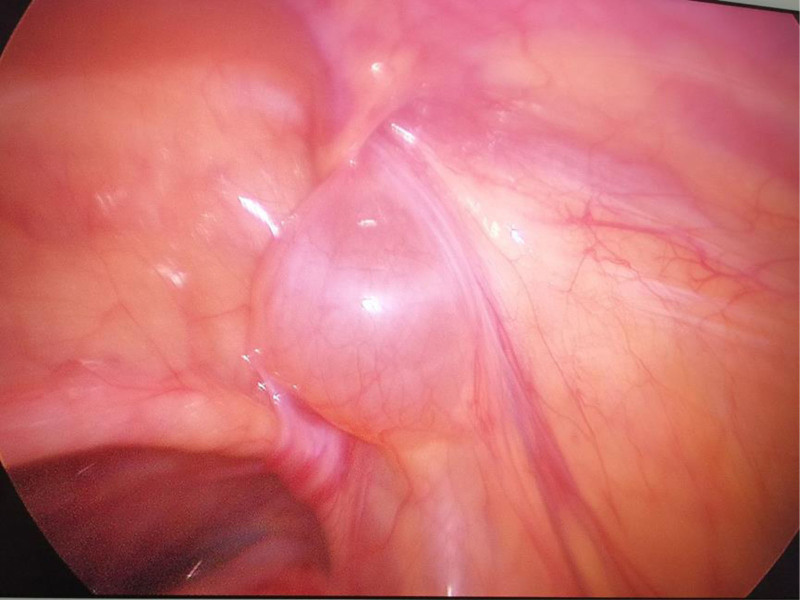
Cyst visible at the mouth of the inner ring.

**Figure 3.**
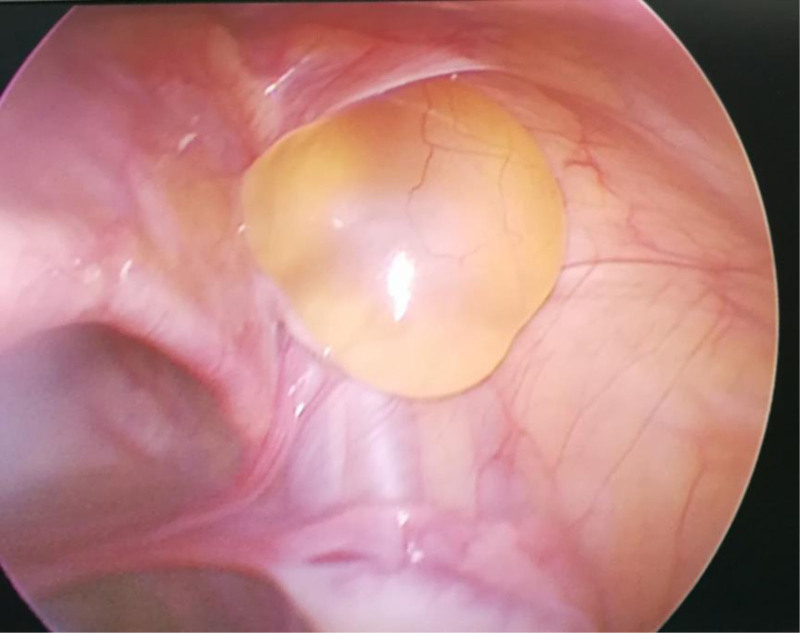
Cyst filling and enlargement at the mouth of the inner ring.

**Figure 4.**
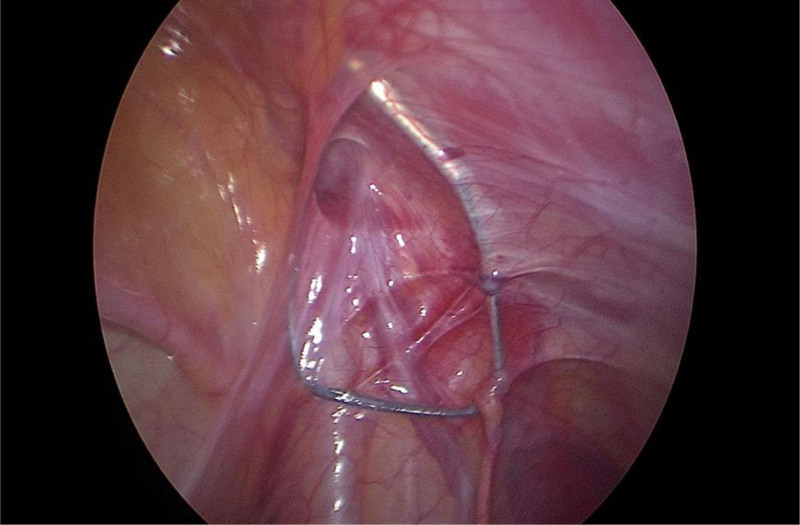
Wide inner annular opening after fluid evacuation.

**Figure 5.**
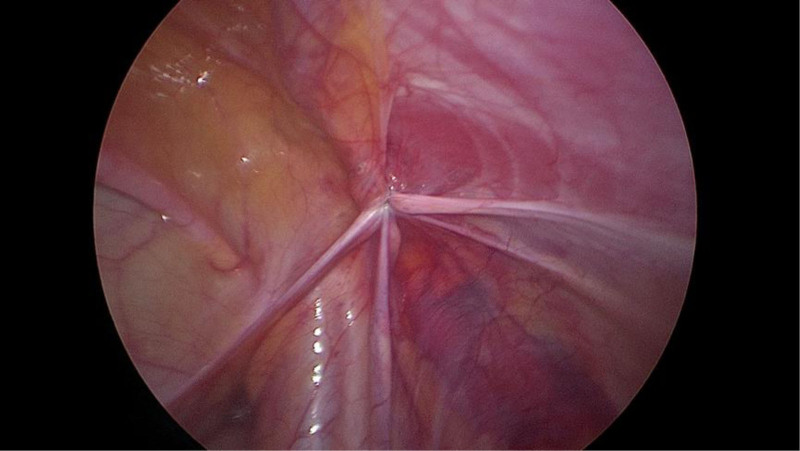
Load suture closure of the affected medial annular opening.

After the success of static-aspiration compound anesthesia, the child was placed flat, and the operation area was disinfected and toweled. A 0.5 cm curved incision was made at the left and right margins of the umbilicus. The abdomen was incised in layers, a 5 mm trocar was punctured, a CO_2_ pneumoperitoneum was established, and the pressure was maintained at 8 mm Hg. The child’s head was lowered by 15°, and a laparoscope was inserted for observation. A cyst was seen in the inner ring on the affected side (Fig. 2), and the cyst was seen to be filling up and becoming more considerable in the inner ring on the affected side when the cyst was extruded from the affected side of the scrotum (Fig. 3). Avoiding the spermatic vessels and vas deferens, the most prominent position of the cyst was opened with tissue scissors to cut the cyst wall extensively. When there was a separation, the separation was cut extensively to ensure that the outflow was emptied after the opening of the cyst. If there was obvious bleeding, the bleeding was stopped by electrocoagulation. After evacuation of the fluid, a vast internal ring opening and unclosed sheath protrusion were seen (Fig. 4); the scrotum was pulled to pull the protruding cystic wall into the internal ring opening; the cystic wall was not treated otherwise, and the intra-abdominal purse-string suture was closed to close the internal ring opening on the affected side and tied with a knot (Fig. 5). After checking that there was no apparent bleeding, counting the instruments and dressings without error, closing the pneumoperitoneum, withdrawing the Trocar and laparoscope, suturing the incision layer by layer, and aspirating the fluid in the scrotum by puncture with a disposable syringe.

## 4. Discussion

Abdominal scrotal hypospadias (ASH) was first described by Dupuytren in 1834 and officially named by Bickle. ASH is a specific type of syringomyelia, clinically characterized by a cystic mass in the inguinal or scrotal area extending through the inguinal canal into the abdominal cavity in the form of a “dumbbell” or “dumbbell-shaped” or “gourd-shaped.”^[3]^ As ASH is relatively rare in clinical practice, the extra-abdominal clinical manifestations and signs are similar to those of conventional syringomyelia, and it is easy to diagnose it as conventional syringomyelia, thus purely performing syringomyelia elevation ligation, which carries the risk of mismanagement and recurrence.^[4]^

The etiology of ASH is not fully understood, but various theories have been proposed. The most widely accepted theory is that over time, a large amount of scrotal fluid passes through the internal inguinal ring into the extraperitoneal space. With the widespread use of ultrasonography, the incidence of ASH has increased in the literature. The diagnosis is suggested by the palpation of a peritoneal effusion that expands and compresses the scrotal portion of the scrotum, called the “bouncing ball sign.” Early surgical intervention is recommended, as spontaneous regression is rare, and the large size of this lesion may lead to testicular malformation, hydrocele and testicular torsion.^[5,6]^ Degeneration into para testicular malignant mesothelioma has also been reported,^[7]^ and although this is rare, it is often considered a factor in the decision to resect the abdominal portion.

There is no uniform conclusion about the pathogenesis of ASH, and there are several main hypotheses: (1) Pressure theory: When the pressure in the sphincter cavity increases, the excessive pressure causes the sphincter capsule to extend into the abdominal cavity through the inguinal canal. The capsule cavity is divided into the intra-abdominal and intrascrotal portions, and the capsule is extruded to become gourd-shaped or dumbbell-shaped at the inguinal canal. (2) Unidirectional flap theory: As the syringomyelia is not closed, the effusion gradually increases. When the pressure is too high, the syringomyelia acts like a 1-way flap counteracting the abdominal cavity, forming the intra-abdominal portion of ASH. Therefore, the inner ring of the inguinal canal is more comprehensive in all children with ASH. (3) Peritoneal diverticulum theory. (4) The theory of extraperitoneal cysts prolapsing through the groin.^[2]^ The first theory is currently more accepted. It is also possible that the occurrence of ASH may result from a multifactorial combination of factors, especially the first and second doctrines. In addition, there are also subperitoneal or extraperitoneal particular types of ASH; Hisamatsu, Takagi, Nomi, et al^[8]^ found that the cysts do not communicate with the mouth of the internal ring, and it is hypothesized that it may be related to the mechanism of plasma hypersecretion or decreased absorption.

The diagnosis and differential diagnosis of ASH is based on an improved understanding of the pathogenesis and clinical manifestations of ASH. The diagnosis can be confirmed by physical examination and ultrasound findings of a cystic mass in the abdominal cavity and scrotum via the inguinal canal. In some cases, the diagnosis is further confirmed by the “bouncing ball sign” in combination with a transillumination test. The development of ultrasonography has further improved the diagnostic rate of ASH, which is characterized by the extension of echogenic cysts in the scrotal spermatic cord through the inguinal canal into the abdominal cavity, with the cysts visible at the mouth of the internal ring and the cysts in the abdominal cavity enlarged by compression of the cysts in the scrotum.^[9]^

Ultrasonography can assess the nature and extent of the scrotal and intra-abdominal cysts and the surrounding tissues, such as testicular development and upper urinary tract. Studies have shown that magnetic resonance imaging or CT with enhancement scanning can help diagnose ASH. Still, they should only be used in patients who cannot be clarified by ultrasonography, considering the higher cost and risk of sedation. Recently, laparoscopic techniques have been widely used in the treatment of syringomyelia in children, with a consequent increase in the number of reported cases of ASH.

Clinical misdiagnosis or underdiagnosis of ASH may lead to the treatment in the wrong area, prolonging the time to confirm the diagnosis, increasing the cost of examination and treatment, and may even increase the surgical trauma due to open surgery for preoperative diagnosis of abdominopelvic tumors. ASH is easily misdiagnosed as retroperitoneal lymphangioma due to the similarity of ultrasound and other imaging findings. Although both are cystic masses, retroperitoneal lymphangiomas often present as multicystic and do not combine with spermatic sheath effusion. Pathology showed that the cystic wall of lymphangioma was covered with flat epithelium, with smooth muscle fibers, blood vessels, fat, lymph, and other tissues and was immunohistochemically positive for D2-40. In contrast, the cystic wall of the ASH abdominal cavity had no epithelial coverage and was immunohistochemically harmful to D2-40. Therefore, intraoperative removal of the complete cyst wall or part of the cyst wall for pathologic examination can also avoid misdiagnosis.

Giant ASH in the pelvis may cause related complications due to compression of the surrounding tissues, and common complications are edema of the lower limbs, pelvic ureteral effusion, relatively rare testicular anomalies, appendicitis, infections, etc. In the present case, the child presented to the clinic with abdominal pain and fever, and there was no obvious abnormality in the appearance of the scrotum. The intra-abdominal mass was detected in the ultrasound. Lymphatic cysts were still considered in the CT intensified examination, and a combined inguinal hernia could not be ruled out. CT examination revealed an open internal ring opening on the same side, which did not exclude the combination of inguinal hernia. However, we still first considered the abdominal lymphatic cyst before the operation and did not think about the possibility of abdominal scrotal syringomyelia because this child’s scrotum did not have an obvious abnormality. It also gave us the wrong guidance. Therefore, clinical attention should be paid to the occurrence of lower extremity edema, hydronephrosis, and inguinal hernia, along with the definitive diagnosis.

The natural course of ASH is unclear, and only a few cases have been reported in which ASH resolves on its own, and only intra-abdominal fluid is absorbed, while intrascrotal fluid persists. Several studies have shown that hydronephrosis, testicular abnormalities, and other pathologic changes, including cryptorchidism, inguinal hernia, and contralateral syringomyelia, may complicate ASH. Therefore, most scholars recommend surgical treatment. With a more in-depth understanding of the disease, surgical approaches to ASH have been improved, reducing the incidence of recurrence and complications. However, there is still controversy about the optimal surgical approach for ASH, as well as inconclusive information about the need for surgical removal of the cysts, the timing of surgery, and the presence of other potential harms. The traditional surgical approach uses complete removal of the cyst via inguinal, scrotal, or combined approaches to prevent the recurrence of ASH. However, extensive surgical excision has been reported, which may disrupt the anatomy of the inguinal canal, vas deferens and spermatic cord injury, and postoperative complications such as scrotal hematoma and testicular atrophy. In this regard, some operators have adopted the surgical method of partial resection of the cystic wall within the inguinal canal to avoid damage to the spermatic cord and vas deferens structures.

Laparoscopic surgery for the treatment of pediatric syringomyelia is less invasive, and experience has been gained in the laparoscopic treatment of ASH in recent years. Current literature mostly resects the cyst, and some operators perform laparoscopic partial resection of the cyst wall. Some scholars found that the child’s syringomyelia was not closed intraoperatively. The surgical method of ligating the syringomyelia was used. The simple closure of the internal ring opening could reduce the damage to the inguinal and scrotal structures and reduce the occurrence of bleeding, testicular atrophy, and other complications. Currently, some operators have used laparoscopic techniques to resect part of the cyst wall while repairing the internal ring orifice and laparoscopic cyst opening combined with high ligation of the sheath protrusion at a single site to treat ASH and have achieved satisfactory results. It is believed that if only the peritoneum or sheath protrusion is ligated and sutured at the internal ring orifice without internal ring repair, the postoperative intra-abdominal pressure is increased. The abdominal cavity contents can still protrude into the inguinal canal through the wide internal ring orifice, and the risk of inguinal hernia recurrence is increased after the operation. The risk of recurrence of inguinal hernia after surgery is increased. In this case, we used laparoscopic exploration, intraoperative diagnosis of abdominal scrotal sheath effusion and exploration of the abdominopelvic cavity; no other lesions were found, and we utilized our previous experience in treating abdominal scrotal sheath effusion. First, the cysts were opened, and then laparoscopic sheath ligation with high ligation was performed, which embodied the advantages of the laparoscopic diagnosis and treatment of ASH. The follow-up of the previous cases did not find any recurrence.

In summary, ASH is a particular type of syringomyelia, which is rare clinically. Clinical symptoms and signs appear similar to conventional syringomyelia, which should be paid attention to in clinical work. The pathogenetic basis for forming intra-abdominal cystic part is still undetermined, and the treatment modality is still not standardized. Ultrasonography is essential for diagnosing ASH; laparoscopic diagnosis and treatment have unique advantages, such as minor injuries and few complications. The treatment of children’s ASH is a minimally invasive, safe and effective surgical modality and is postoperative effect good; at the same time, intraoperative pathological biopsy to avoid missed diagnosis and misdiagnosis. Through this case, we still need to raise awareness of ASH, especially for children who have no prominent appearance of scrotal inguinal manifestations or who come to the clinic with other possible comorbidities; we should think about the possibility of ASH to achieve a rapid diagnosis, choose the proper treatment, reduce costs and trauma, and avoid potential risks.

## Author contributions

**Conceptualization:** Ti-Xue Wang, Lin Feng.

**Data curation:** Ti-Xue Wang.

**Investigation:** Ya-Long Ma.

**Methodology:** Ti-Xue Wang, Lin Feng.

**Validation:** Nuan Han, Chong-Fang Zhang.

**Visualization:** Ya-Long Ma.

**Writing – original draft:** Ti-Xue Wang.

**Writing – review & editing:** Lin Feng.
